# Glycyrrhizin for treatment of CRS caused by CAR T-cell therapy: A pharmacological perspective

**DOI:** 10.3389/fphar.2023.1134174

**Published:** 2023-02-27

**Authors:** Xingxing Qi, Juan Li, Pan Luo

**Affiliations:** Department of Pharmacy, Tongji Hospital, Tongji Medical College, Huazhong University of Science and Technology, Wuhan, China

**Keywords:** glycyrrhizin, chimeric antigen receptor T-cell therapy, cytokine release syndrome, tocilizumab, corticosteroids

## Abstract

Chimeric antigen receptor T (CAR T)-cell therapy promises to revolutionize the management of hematologic malignancies and possibly other tumors. However, the main side effect of cytokine release syndrome (CRS) is a great challenge for its clinical application. Currently, treatment of CRS caused by CAR T-cell therapy is limited to tocilizumab (TCZ) and corticosteroids in clinical guidelines. However, the theoretical risks of these two agents may curb clinicians’ enthusiasm for their application, and the optimal treatment is still debated. CAR T-cell therapy induced-CRS treatment is a current research focus. Glycyrrhizin, which has diverse pharmacological effects, good tolerance, and affordability, is an ideal therapeutic alternative for CRS. It can also overcome the shortcoming of TCZ and corticosteroids. In this brief article, we discuss the therapeutic potential of glycyrrhizin for treating CRS caused by CAR T-cell therapy from the perspective of its pharmacological action.

## 1 Introduction

Chimeric antigen receptor T (CAR T) cells, in the simplest form, are T cells that are genetically engineered with a CAR structure that can recognize a specific antigen on the tumor cell surface and destroy malignant cells. Over the past decades, significant progress has been made in the development of CAR T-cell therapy. This therapy has initiated a new class of therapies and gained fresh prominence for antitumor treatment, especially in hematologic malignancies. Today, CAR T-cell therapy is a therapy that can cure patients with certain hematologic malignancies, although oncologists have not used the word “cure” lightly (Freyer and Porter, 2020).

One of the major hallmark challenges associated with almost all CAR T-cell therapies is the development of cytokine release syndrome (CRS). CRS is a potentially life-threatening systemic inflammatory response driven by elevations in inflammatory cytokines and chemokines. It is characterized by flu-like symptoms, hypotension, hypoxia, and even multi-organ failure in severe cases. The onset latency of CRS is determined by a multitude of factors, and CRS usually occurs within the first 2 weeks after CAR T-cell administration. Currently, only corticosteroids and tocilizumab (TCZ, an IL-6 receptor antagonist that blocks IL-6-mediated signal transduction by inhibiting IL-6 binding to IL-6 receptor and is the only FDA-approved therapy for treating CART-cell-associated CRS (Si and Teachey, 2020)) are recommended by many national and international guidelines for the management of CRS (Santomasso et al., 2021; Hayden et al., 2022). However, the theoretical risks of impairing the function of CAR T cells with the use of corticosteroids, worsening neurotoxicity with the use of TCZ, and predisposing patients to infections with the use of either agents may tend to stifle clinicians’ initiative to prescribe these two drugs though data supporting these risks are relatively limited (Banerjee et al., 2021). Contradictory evidence of the lethal effect of corticosteroids on CAR T cells has been found in different studies (Brentjens et al., 2013; Davila et al., 2014; Liu et al., 2020a). Nevertheless, it has been demonstrated in real-world analyses that higher cumulative doses of corticosteroids exposure, as well as both prolonged and early use of corticosteroids, were associated with worse overall survival, following CAR T-cell therapy (Strati et al., 2021), suggesting that the risk of corticosteroids affecting the amplification and persistence of CAR T cells is real. IL-6 is mainly eliminated *via* IL-6 receptor-mediated clearance, and TCZ treatment can induce a marked increase in serum IL-6 (Nishimoto et al., 2008). The transient rise of serum IL-6 can increase the passive diffusion of IL-6 into cerebrospinal fluid (CSF), but TCZ cannot cross the blood–brain barrier as easily as IL-6 (Nellan et al., 2018). Theoretically, TCZ could exacerbate cytokine-mediated neurotoxicity by causing an unopposed increase of IL-6 in CSF. This hypothesis is supported by the fact that patients who received TCZ treatment for CAR T-cell-related CRS were more likely to experience neurotoxicity (Frigault et al., 2020). IL-6 is a pivotal cytokine in the integrated immune response. One of its roles is to support the host in responding to infections (Rose-John et al., 2017). Hence, IL-6 receptor antagonist TCZ treatment might increase the risk of infections, as has been generally observed in corticosteroid therapies. Corticosteroid therapies can induce complicated infections by suppressing the immune response and altering host defense (Dale and Petersdorf, 1973). Although some data suggest that short-term use of TCZ for CRS will not significantly increase the susceptibility to infectious complications (Frigault et al., 2020), the use of corticosteroids is associated with higher rates of infections (Baird et al., 2021). More importantly, treatment of CRS with TCZ is clinically ineffective in more than 30% of patients, and corticosteroid-refractory CRS can also develop in some patients (Pan et al., 2021). Therefore, a novel treatment to minimize the lethal severity of CRS and maximize the benefits associated with CAR T-cell therapy is urgently needed in the clinical setting.

## 2 Glycyrrhizin and its potential advantages in the treatment of CRS induced by CAR T-cell therapy

Glycyrrhizin, also known as glycyrrhizinic acid, is a triterpene glycoside (saponin) with a molecular formula of C_42_H_62_O_16_ and a weight of 823 g/mol. It is the main water-soluble component of licorice root extract and consists of one molecule of glycyrrhetinic acid and two molecules of glucuronic acid. Glycyrrhizin has been used in China for more than 4,000 years. It is widely employed to treat a variety of diseases and conditions because it possesses multiple pharmacological properties, including anti-inflammation, antioxidative, immunomodulatory, antiviral, anticancer, and hepatoprotective effects (Chen et al., 2020; Rehman et al., 2020; Bakr et al., 2022).

**FIGURE 1:**
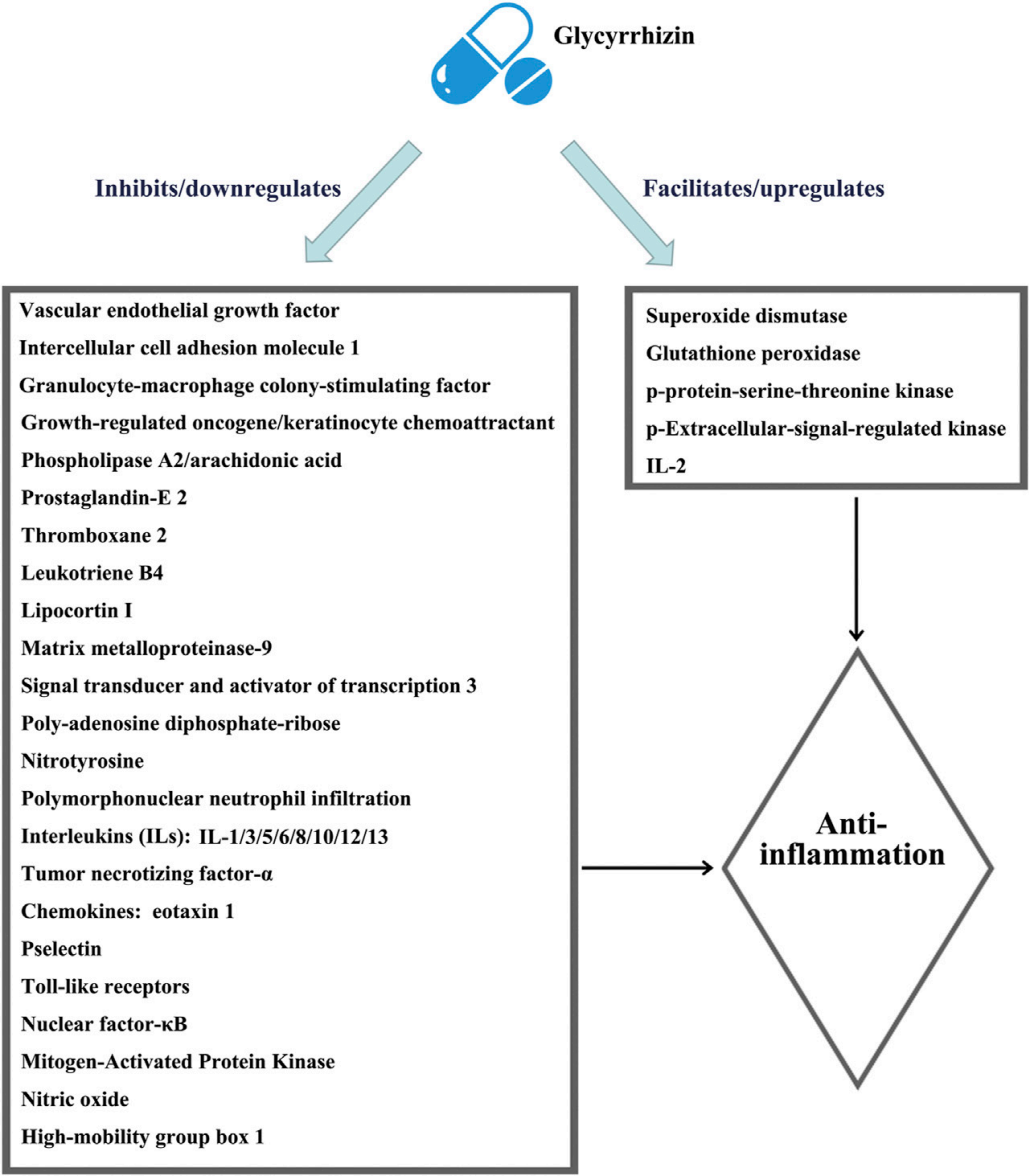
Schematic illustration of the comprehensive anti-inflammatory mechanisms of glycyrrhizin. The figure is drawn based on Figure 1 of Richard (2021).

The most important pharmacological effect of glycyrrhizin is anti-inflammation, and its anti-inflammatory actions are similar to those of glucocorticoids due to the structural similarity of glycyrrhizin with adrenocortical hormones (Chen et al., 2020). Glycyrrhizin elicits broad-spectrum anti-inflammatory actions *via* interacting with various inflammatory factors and pathways, as shown in Figure 1, which were summarized in detail by Richard (2021). However, unlike glucocorticoids that elicit immune-suppressing effects, glycyrrhizin is expected to enhance the immune response (Li et al., 2011; Soufy et al., 2012; Xu et al., 2018a). Moreover, unlike glucocorticoids that preferentially affect T lymphocytes for rapid apoptotic cell death (Tuosto et al., 1994), glycyrrhizin displays a mild action to slowly induce the apoptotic death of lymphocytes (Oh et al., 1999).

Some studies suggested that glycyrrhizin can stimulate both T- and B-lymphocyte proliferation (Chavali et al., 1987; Jiang et al., 2020). Compared with glucocorticoids, it seems that the negative impact of glycyrrhizin on the pharmacokinetics/survival of CAR T cells is extremely low, and administration of glycyrrhizin will not put the patient into a condition with a risk of infections. In addition, with its broad-spectrum anti-inflammatory properties, glycyrrhizin can minimize the rate of clinical inefficacy when managing CRS, and it should be more effective than the therapies like TCZ that only target one single cytokine. No evidence indicates that glycyrrhizin can worsen neurotoxicity. Therefore, as expected from the established role of glycyrrhizin, glycyrrhizin could be a promising treatment for CRS with some distinct advantages over glucocorticoids and TCZ.

## 3 Broad-spectrum anti-inflammatory capability forms the cornerstone of glycyrrhizin in managing CRS induced by CAR T-cell therapy

**FIGURE 2:**
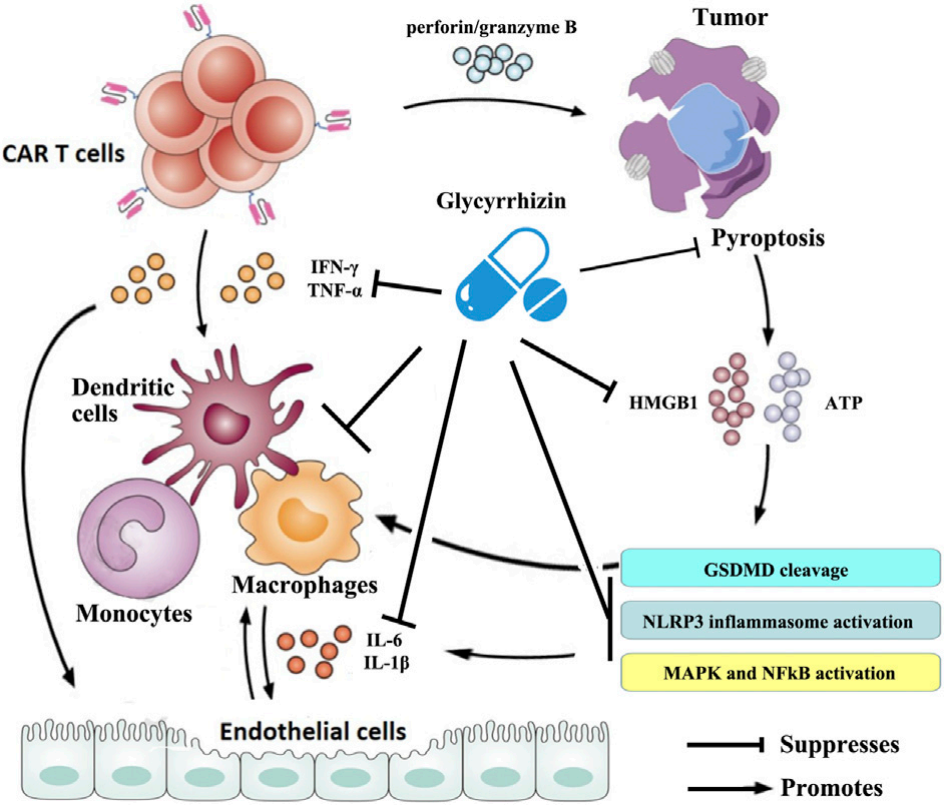
Schematic illustration of the therapeutic benefits of glycyrrhizin for CRS triggered by CAR T-cell therapy. The figure is drawn based on Figure 1 of Schubert et al. (2021) and Xiao et al. (2021).

In recent years, great progress has been made in exploring the potential pathophysiology of CRS trigged by CAR T-cell therapies. It is now generally accepted that CAR T cells are activated following target tumor cells and then induce the release of various inflammatory factors such as IFN-γ, TNF-α, which leads to the activation of bystander myeloid cell populations (e.g., monocytes, macrophages, and dendritic cells) and endothelial cells. These cells further promote the rapid production and secretion of proinflammatory cytokines such as IL-6 and IL-1β that trigger a cascade reaction and contribute to inflammatory toxicities. Large amounts of IL-6, in turn, activate the T cells and other immune cells and then lead to a positive CRS feedback loop (Cobb and Lee, 2021; Cosenza et al., 2021; Schubert et al., 2021). Therefore, strategies targeted at the cytokines mentioned previously or that can reduce myeloid and/or endothelial cell activation may prevent CRS. Numerous studies have shown that glycyrrhizin can downregulate the mRNA expression and the production of cytokines such as IL-1β, IL-6, TNF-α, and IFN-γ (Ni et al., 2011; Chen et al., 2017; Yu et al., 2017; Xu et al., 2018a; Sun et al., 2018; Tian et al., 2019) and mitigate the activation or dysfunction of monocytes, macrophages, dendritic cells, and endothelial cells in a variety of pathological models (Matsushima and Baba, 1992; Feng et al., 2013; Wakabayashi et al., 2018; Gowda et al., 2021). Some clinical trials have confirmed the anti-inflammatory properties of glycyrrhizin (Takeuchi-Hatanaka et al., 2016; Li et al., 2019; Cao et al., 2020). On a broader aspect, glycyrrhizin can represent an excellent therapeutic modality for CRS triggered by CAR T-cell therapies (Figure 2).

## 4 Suppressing pyroptosis contributes to glycyrrhizin’s potential therapeutic effects in managing CRS induced by CAR T-cell therapy

Recent research conducted by Liu *et al.* showed that CAR T cells rapidly activate caspase-3 in human B leukemic cells and other targeted tumor cells through releasing of perforin/granzyme B and leading to pyroptosis of the targeted cells. Consequently, pyroptosis-released factors activate caspase-1 for gasdermin D (GSDMD) cleavage in macrophages to stimulate macrophages to produce proinflammatory cytokines, which may trigger CRS in CAR T-cell-treated patients (Liu et al., 2020b). However, it was reported that glycyrrhizin plays a role in inhibiting caspase 1/GSDMD and suppressing pyroptosis (Hua et al., 2019; Wang et al., 2020), indicating that it could block the occurrence of CRS in CAR T-cell treated patients. Moreover, the results from Liu *et al.* demonstrated that pyroptosis-released factors, particularly extensive extracellular adenosine 5′-triphosphate (ATP) and high-mobility group box 1 (HMGB1), contribute to the release of the CRS-related cytokine by macrophages (Liu et al., 2020a). ATP activates NACHT, LRR, and the PYD domain-containing protein 3 (NLRP3) inflammasome that cleaves caspase-1 in macrophages to promote the release of IL-1β. HMGB1 may induce IL-6 production in macrophages after tumor cell pyroptosis through the activation of mitogen-activated protein kinase (MAPK) and nuclear factor κB (NF-κB) (Liu et al., 2020b). It has been observed that glycyrrhizin has efficacy in suppressing the NLRP3 inflammasome and inhibiting the activation of NF-κB and MAPK signaling pathways (Yao and Sun, 2019). More importantly, glycyrrhizin itself was frequently used as an inhibitor of HMGB1. Research has also confirmed that glycyrrhizin can rescue macrophage activation induced by multiple etiological factors (Li et al., 2015; Gowda et al., 2021). These evidences suggest that glycyrrhizin has a potential effect in regulating CRS during CAR T-cell therapy by intervening in pyroptosis and/or its cascades (Figure 2).

## 5 Targeting multiple intracellular signaling pathways contributes to the potential therapeutic effects of glycyrrhizin in managing CRS induced by CAR T-cell therapy

Several studies have found that the use of small molecule inhibitors, including ruxolitinib (an inhibitor of Janus kinases 1 and 2) and itacitinib (a selective Janus kinase 1 inhibitor), may also help prevent CRS induced by CAR T-cell therapy (Huarte et al., 2020; Pan et al., 2021; Xu et al., 2022). Strikingly, it was identified that glycyrrhizin could inhibit the phosphorylation of Janus kinases 1 and 2 and reduce the activity of the JAK/STAT signaling pathway (Xu et al., 2018b; Wu et al., 2018). Moreover, recent studies have confirmed that dasatinib can switch off the cytokine release to reduce the risk of CRS by inhibiting the SRC family kinase lymphocyte-specific protein tyrosine kinase (LCK) (Mestermann et al., 2019; Leclercq et al., 2021). SRC family kinases share a common architecture that underlies a shared regulatory mechanism (Sicheri and Kuriyan, 1997). It should be noted that glycyrrhizin could decrease SRC kinase activity reported in a previous study (Wu et al., 2015), suggesting that it may have a similar capacity of effectively blocking CRS to dasatinib. These data indicate that glycyrrhizin holds a great developmental and application prospect in the treatment of CRS caused by CAR T-cell therapy.

## 6 Other potential therapeutic benefits of glycyrrhizin in CAR T-cell therapy

Glycyrrhizin, owing to its antipyretic action, hypertension activity, and inhibitory effect on airway mucus hyperproduction (Yanagawa et al., 2004; Nishimoto et al., 2010; Nazari et al., 2017), could be used as a supportive remedy for the fever, hypotension, and hypoxia that are the manifestations of CRS. In addition, adverse events, including immune effector cell-associated neurotoxicity syndrome (ICANS)/neurotoxicity and opportunistic infections, are common in CAR T-cell therapy, and they are associated with CRS (Hill et al., 2018; Ruff et al., 2020). Overwhelming evidence affirms that glycyrrhizin has beneficial antiviral, antibacterial, antifungal, and neuroprotective activities (Huan et al., 2021; Astaf’eva and Sukhenko, 2014; Utsunomiya et al., 1999; Paudel et al., 2020). These activities make glycyrrhizin a potential agent for adjuvant or preventive therapy in simultaneously managing other side effects of CAR T-cell therapy.

It is a general consensus that glycyrrhizin has broad activity against a wide variety of tumor cell types (Roohbakhsh et al., 2016). Furthermore, previous reports showed that glycyrrhizin was tolerated by normal human leukocytes/peripheral blood mononuclear cells, but it was effective in the treatment of both chronic myeloid leukemia and lymphoma *in vitro* or *in vivo* (Hibasami et al., 2006; Hostetler et al., 2017). These make glycyrrhizin an attractive agent for combinational therapy with CAR T cells in hematological malignancies, raising its value beyond CRS management. It has been identified that adding a programmed cell death protein-1 (PD-1) blockade to CAR T-cell therapy can escalate CAR T-cell function and, to some extent, improve prognosis and efficacy (Song and Zhang, 2020). However, it has been reported that PD-L1/PD-1 upregulation can be mediated by autocrine and paracrine activation of HMGB1 signaling (Wang et al., 2019; Xu et al., 2021). Glycyrrhizin, a direct inhibitor of HMGB1, may hold promise for a similar job as PD-1 blockade when combined with CAR T cells, reaffirming the superiority of using glycyrrhizin in CAR T-cell therapy.

## 7 Discussion

The optimal treatment regimen of CRS caused by CAR T-cell therapy is still a matter of debate and not well-defined. Glycyrrhizin is valued for its many pharmacological effects discussed previously, which make it a promising therapeutic alternative. It can overcome the shortcomings of the current mainstream therapeutic strategies (corticosteroids and TCZ) against CRS. Glycyrrhizin can inhibit almost all factors responsible for inflammatory reactions (Rehman et al., 2020). This is a decisive advantage over alternative strategies in CRS management, including those that can only block or neutralize IL-1, IL-6, and GM-CSF. That glycyrrhizin is safe, tolerable, convenient, and affordable adds interest to its clinical application. Therefore, glycyrrhizin can be preemptively or even prophylactically used in the early-grade CRS and can be administered for a longer course. However, the conjecture we proposed is based on the extensive pharmacological properties of glycyrrhizin. The clinical efficacy and safety and appropriate dosage regimens of glycyrrhizin in real-life clinical scenarios need to be further investigated with well-designed clinical trials.

## Data Availability

The original contributions presented in the study are included in the article/Supplementary Material; further inquiries can be directed to the corresponding authors.

## References

[B1] Astaf'eva O. V. Sukhenko L. T. (2014). Comparative analysis of antibacterial properties and chemical composition of Glycyrrhiza glabra L. from Astrakhan region (Russia) and Calabria region (Italy). Bull. Exp. Biol. Med. 156 (6), 829–832. 10.1007/s10517-014-2462-8 24824709

[B2] Baird J. H. Epstein D. J. Tamaresis J. S. Ehlinger Z. Spiegel J. Y. Craig J. (2021). Immune reconstitution and infectious complications following axicabtagene ciloleucel therapy for large B-cell lymphoma. Blood Adv. 5 (1), 143–155. 10.1182/bloodadvances.2020002732 33570626 PMC7805341

[B3] Bakr A. F. Shao P. Farag M. A. (2022). Recent advances in glycyrrhizin metabolism, health benefits, clinical effects and drug delivery systems for efficacy improvement; a comprehensive review. Phytomedicine 99, 153999. 10.1016/j.phymed.2022.153999 35220130

[B4] Banerjee R. Fakhri B. Shah N. (2021). Toci or not toci: Innovations in the diagnosis, prevention, and early management of cytokine release syndrome. Leuk. Lymphoma 62, 2600–2611. 10.1080/10428194.2021.1924370 34151714

[B5] Brentjens R. J. Davila M. L. Riviere I. Park J. Wang X. Cowell L. G. (2013). CD19-targeted T cells rapidly induce molecular remissions in adults with chemotherapy-refractory acute lymphoblastic leukemia. Sci. Transl. Med. 5 (177), 177ra38. 10.1126/scitranslmed.3005930 PMC3742551 23515080

[B6] Cao Z. Y. Liu Y. Z. Li J. M. Ruan Y. M. Yan W. J. Zhong S. Y. (2020). Glycyrrhizic acid as an adjunctive treatment for depression through anti-inflammation: A randomized placebo-controlled clinical trial. J. Affect Disord. 265, 247–254. 10.1016/j.jad.2020.01.048 32090748

[B7] Chavali S. R. Francis T. Campbell J. B. (1987). An *in vitro* study of immunomodulatory effects of some saponins. Int. J. Immunopharmacol. 9 (6), 675–683. 10.1016/0192-0561(87)90038-5 3500923

[B8] Chen K. Yang R. Shen F. Q. Zhu H. L. (2020). Advances in pharmacological activities and mechanisms of glycyrrhizic acid. Curr. Med. Chem. 27 (36), 6219–6243. 10.2174/0929867325666191011115407 31612817

[B9] Chen X. Fang D. Li L. Chen L. Li Q. Gong F. (2017). Glycyrrhizin ameliorates experimental colitis through attenuating interleukin-17-producing T cell responses via regulating antigen-presenting cells. Immunol. Res. 65 (3), 666–680. 10.1007/s12026-017-8894-2 28108937

[B10] Cobb D. A. Lee D. W. (2021). Cytokine release syndrome biology and management. Cancer J. 27 (2), 119–125. 10.1097/PPO.0000000000000515 33750071

[B11] Cosenza M. Sacchi S. Pozzi S. (2021). Cytokine release syndrome associated with T-cell-based therapies for hematological malignancies: Pathophysiology, clinical presentation, and treatment. Int. J. Mol. Sci. 22 (14), 7652. 10.3390/ijms22147652 34299273 PMC8305850

[B12] Dale D. C. Petersdorf R. G. (1973). Corticosteroids and infectious diseases. Med. Clin. North Am. 57 (5), 1277–1287. 10.1016/s0025-7125(16)32228-3 4593200

[B13] Davila M. L. Riviere I. Wang X. Bartido S. Park J. Curran K. (2014). Efficacy and toxicity management of 19-28z CAR T cell therapy in B cell acute lymphoblastic leukemia. Sci. Transl. Med. 6 (224), 224ra25. 10.1126/scitranslmed.3008226 PMC4684949 24553386

[B14] Feng L. Zhu M. M. Zhang M. H. Wang R. S. Tan X. B. Song J. (2013). Protection of glycyrrhizic acid against AGEs-induced endothelial dysfunction through inhibiting RAGE/NF-κB pathway activation in human umbilical vein endothelial cells. J. Ethnopharmacol. 148 (1), 27–36. 10.1016/j.jep.2013.03.035 23528363

[B15] Freyer C. W. Porter D. L. (2020). Advances in CAR T therapy for hematologic malignancies. Pharmacotherapy 40 (8), 741–755. 10.1002/phar.2414 32383222

[B16] Frigault M. J. Nikiforow S. Mansour M. K. Hu Z. H. Horowitz M. M. Riches M. L. (2020). Tocilizumab not associated with increased infection risk after CAR T-cell therapy: Implications for COVID-19? Blood 136 (1), 137–139. 10.1182/blood.2020006216 32457999 PMC7332891

[B17] Gowda P. Patrick S. Joshi S. D. Kumawat R. K. Sen E. (2021). Glycyrrhizin prevents SARS-CoV-2 S1 and Orf3a induced high mobility group box 1 (HMGB1) release and inhibits viral replication. Cytokine 142, 155496. 10.1016/j.cyto.2021.155496 33773396 PMC7953444

[B18] Hayden P. J. Roddie C. Bader P. Basak G. W. Bonig H. Bonini C. (2022). Management of adults and children receiving CAR T-cell therapy: 2021 best practice recommendations of the European society for blood and marrow transplantation (EBMT) and the joint accreditation committee of ISCT and EBMT (JACIE) and the European haematology association (EHA). Ann. Oncol. 33 (3), 259–275. 10.1016/j.annonc.2021.12.003 34923107

[B19] Hibasami H. Iwase H. Yoshioka K. Takahashi H. (2006). Glycyrrhetic acid (a metabolic substance and aglycon of glycyrrhizin) induces apoptosis in human hepatoma, promyelotic leukemia and stomach cancer cells. Int. J. Mol. Med. 17 (2), 215–219. 10.3892/ijmm.17.2.215 16391818

[B20] Hill J. A. Li D. Hay K. A. Green M. L. Cherian S. Chen X. (2018). Infectious complications of CD19-targeted chimeric antigen receptor-modified T-cell immunotherapy. Blood 131 (1), 121–130. 10.1182/blood-2017-07-793760 29038338 PMC5755046

[B21] Hostetler B. J. Uchakina O. N. Ban H. McKallip R. J. (2017). Treatment of hematological malignancies with glycyrrhizic acid. Anticancer Res. 37 (3), 997–1004. 10.21873/anticanres.11409 28314257

[B22] Hua S. Ma M. Fei X. Zhang Y. Gong F. Fang M. (2019). Glycyrrhizin attenuates hepatic ischemia-reperfusion injury by suppressing HMGB1-dependent GSDMD-mediated kupffer cells pyroptosis. Int. Immunopharmacol. 68, 145–155. 10.1016/j.intimp.2019.01.002 30634142

[B23] Huan C. Xu Y. Zhang W. Guo T. Pan H. Gao S. (2021). Research progress on the antiviral activity of glycyrrhizin and its derivatives in liquorice. Front. Pharmacol. 12, 680674. 10.3389/fphar.2021.680674 34295250 PMC8290359

[B24] Huarte E. O'Connor R. S. Peel M. T. Nunez-Cruz S. Leferovich J. Juvekar A. (2020). Itacitinib (INCB039110), a JAK1 inhibitor, reduces cytokines associated with cytokine release syndrome induced by CAR T-cell therapy. Clin. Cancer Res. 26 (23), 6299–6309. 10.1158/1078-0432.CCR-20-1739 32998963 PMC7895329

[B25] Jiang R. Gao J. Shen J. Zhu X. Wang H. Feng S. (2020). Glycyrrhizic acid improves cognitive levels of aging mice by regulating T/B cell proliferation. Front. Aging Neurosci. 12, 570116. 10.3389/fnagi.2020.570116 33132898 PMC7575738

[B26] Leclercq G. Haegel H. Schneider A. Giusti A. M. Marrer-Berger E. Boetsch C. (2021). Src/lck inhibitor dasatinib reversibly switches off cytokine release and T cell cytotoxicity following stimulation with T cell bispecific antibodies. J. Immunother. Cancer 9 (7), e002582. 10.1136/jitc-2021-002582 34326166 PMC8323395

[B27] Li L. Ma Q. Li H. (2019). Effect of vitiligo treatment using compound glycyrrhizin combined with fractional carbon dioxide laser and topical triamcinolone acetonide on serum interleukin-17 and tissue growth factor-**β** levels. J. Int. Med. Res. 47 (11), 5623–5631. 10.1177/0300060519871382 31550958 PMC6862872

[B28] Li X. L. Zhou A. G. Zhang L. Chen W. J. (2011). Antioxidant status and immune activity of glycyrrhizin in allergic rhinitis mice. Int. J. Mol. Sci. 12 (2), 905–916. 10.3390/ijms12020905 21541033 PMC3083680

[B29] Li X. Yue Y. Zhu Y. Xiong S. (2015). Extracellular, but not intracellular HMGB1, facilitates self-DNA induced macrophage activation via promoting DNA accumulation in endosomes and contributes to the pathogenesis of lupus nephritis. Mol. Immunol. 65 (1), 177–188. 10.1016/j.molimm.2015.01.023 25660970

[B30] Liu S. Deng B. Yin Z. Pan J. Lin Y. Ling Z. (2020a). Corticosteroids do not influence the efficacy and kinetics of CAR T cells for B-cell acute lymphoblastic leukemia. Blood Cancer J. 10 (2), 15. 10.1038/s41408-020-0280-y 32029707 PMC7005173

[B31] Liu S. Fang Y. Chen X. Wang Z. Liang X. Zhang T. (2020b). Gasdermin E-mediated target cell pyroptosis by CAR T cells triggers cytokine release syndrome. Sci. Immunol. 5 (43), eaax7969. 10.1126/sciimmunol.aax7969 31953257

[B32] Matsushima Y. Baba T. (1992). An antigranulomatous effect of glycyrrhizin. J. Exp. Pathol. 6 (1-2), 25–30. 1625037

[B33] Mestermann K. Giavridis T. Weber J. Rydzek J. Frenz S. Nerreter T. (2019). The tyrosine kinase inhibitor dasatinib acts as a pharmacologic on/off switch for CAR T cells. Sci. Transl. Med. 11 (499), eaau5907. 10.1126/scitranslmed.aau5907 31270272 PMC7523030

[B34] Nazari S. Rameshrad M. Hosseinzadeh H. (2017). Toxicological effects of Glycyrrhiza glabra (licorice): A review. Phytother. Res. 31 (11), 1635–1650. 10.1002/ptr.5893 28833680

[B35] Nellan A. McCully C. M. L. Cruz Garcia R. Jayaprakash N. Widemann B. C. Lee D. W. (2018). Improved CNS exposure to tocilizumab after cerebrospinal fluid compared to intravenous administration in rhesus macaques. Blood 132 (6), 662–666. 10.1182/blood-2018-05-846428 29954750 PMC6086204

[B36] Ni Y. F. Kuai J. K. Lu Z. F. Yang G. D. Fu H. Y. Wang J. (2011). Glycyrrhizin treatment is associated with attenuation of lipopolysaccharide-induced acute lung injury by inhibiting cyclooxygenase-2 and inducible nitric oxide synthase expression. J. Surg. Res. 165 (1), e29–e35. 10.1016/j.jss.2010.10.004 21074783

[B37] Nishimoto N. Terao K. Mima T. Nakahara H. Takagi N. Kakehi T. (2008). Mechanisms and pathologic significances in increase in serum interleukin-6 (IL-6) and soluble IL-6 receptor after administration of an anti-IL-6 receptor antibody, tocilizumab, in patients with rheumatoid arthritis and Castleman disease. Blood 112 (10), 3959–3964. 10.1182/blood-2008-05-155846 18784373

[B38] Nishimoto Y. Hisatsune A. Katsuki H. Miyata T. Yokomizo K. Isohama Y. (2010). Glycyrrhizin attenuates mucus production by inhibition of MUC5AC mRNA expression *in vivo* and *in vitro* . J. Pharmacol. Sci. 113 (1), 76–83. 10.1254/jphs.09344fp 20453436

[B39] Oh C. Kim Y. Eun J. Yokoyama T. Kato M. Nakashima I. (1999). Induction of T lymphocyte apoptosis by treatment with glycyrrhizin. Am. J. Chin. Med. 27 (2), 217–226. 10.1142/S0192415X99000252 10467455

[B40] Pan J. Deng B. Ling Z. Song W. Xu J. Duan J. (2021). Ruxolitinib mitigates steroid-refractory CRS during CAR T therapy. J. Cell Mol. Med. 25 (2), 1089–1099. 10.1111/jcmm.16176 33314568 PMC7812291

[B41] Paudel Y. N. Angelopoulou E. Semple B. Piperi C. Othman I. Shaikh M. F. (2020). Potential neuroprotective effect of the HMGB1 inhibitor glycyrrhizin in neurological disorders. ACS Chem. Neurosci. 11 (4), 485–500. 10.1021/acschemneuro.9b00640 31972087

[B42] Rehman M. U. Farooq A. Ali R. Bashir S. Bashir N. Majeed S. (2020). Preclinical evidence for the pharmacological actions of glycyrrhizic acid: A comprehensive review. Curr. Drug Metab. 21 (6), 436–465. 10.2174/1389200221666200620204914 32562521

[B43] Richard S. A. (2021). Exploring the pivotal immunomodulatory and anti-inflammatory potentials of glycyrrhizic and glycyrrhetinic acids. Mediat. Inflamm. 2021, 6699560. 10.1155/2021/6699560 PMC7808814 33505216

[B44] Roohbakhsh A. Iranshahy M. Iranshahi M. (2016). Glycyrrhetinic acid and its derivatives: Anti-cancer and cancer chemopreventive properties, mechanisms of action and structure- cytotoxic activity relationship. Curr. Med. Chem. 23 (5), 498–517. 10.2174/0929867323666160112122256 26758798

[B45] Rose-John S. Winthrop K. Calabrese L. (2017). The role of IL-6 in host defence against infections: Immunobiology and clinical implications. Nat. Rev. Rheumatol. 13 (7), 399–409. 10.1038/nrrheum.2017.83 28615731

[B46] Ruff M. W. Siegler E. L. Kenderian S. S. (2020). A concise review of neurologic complications associated with chimeric antigen receptor T-cell immunotherapy. Neurol. Clin. 38 (4), 953–963. 10.1016/j.ncl.2020.08.001 33040871

[B47] Santomasso B. D. Nastoupil L. J. Adkins S. Lacchetti C. Schneider B. J. Anadkat M. (2021). Management of immune-related adverse events in patients treated with chimeric antigen receptor T-cell therapy: ASCO guideline. J. Clin. Oncol. 39 (35), 3978–3992. 10.1200/JCO.21.01992 34724386

[B48] Schubert M. L. Schmitt M. Wang L. Ramos C. A. Jordan K. Muller-Tidow C. (2021). Side-effect management of chimeric antigen receptor (CAR) T-cell therapy. Ann. Oncol. 32 (1), 34–48. 10.1016/j.annonc.2020.10.478 33098993

[B49] Si S. Teachey D. T. (2020). Spotlight on tocilizumab in the treatment of CAR-T-cell-induced cytokine release syndrome: Clinical evidence to date. Ther. Clin. Risk Manag. 16, 705–714. 10.2147/TCRM.S223468 32801727 PMC7414980

[B50] Sicheri F. Kuriyan J. (1997). Structures of Src-family tyrosine kinases. Curr. Opin. Struct. Biol. 7 (6), 777–785. 10.1016/s0959-440x(97)80146-7 9434895

[B51] Song W. Zhang M. (2020). Use of CAR T cell therapy, PD-1 blockade, and their combination for the treatment of hematological malignancies. Clin. Immunol. 214, 108382. 10.1016/j.clim.2020.108382 32169439

[B52] Soufy H. Yassein S. Ahmed A. R. Khodier M. H. Kutkat M. A. Nasr S. M. (2012). Antiviral and immune stimulant activities of glycyrrhizin against duck hepatitis virus. Afr. J. Tradit. Complement. Altern. Med. 9 (3), 389–395. 10.4314/ajtcam.v9i3.14 23983372 PMC3746675

[B53] Strati P. Ahmed S. Furqan F. Fayad L. E. Lee H. J. Iyer S. P. (2021). Prognostic impact of corticosteroids on efficacy of chimeric antigen receptor T-cell therapy in large B-cell lymphoma. Blood 137 (23), 3272–3276. 10.1182/blood.2020008865 33534891 PMC8351896

[B54] Sun Y. Chen H. Dai J. Wan Z. Xiong P. Xu Y. (2018). Glycyrrhizin protects mice against experimental autoimmune encephalomyelitis by inhibiting high-mobility group box 1 (HMGB1) expression and neuronal HMGB1 release. Front. Immunol. 9, 1518. 10.3389/fimmu.2018.01518 30013568 PMC6036111

[B55] Takeuchi-Hatanaka K. Yasuda T. Naruishi K. Katsuragi-Fuke K. Inubushi J. Ootsuki H. (2016). Effects of new over-the-counter periodontal ointment-containing applicator with single-tuft brush on cytokine levels in gingival crevicular fluid during supportive periodontal therapy phase: A randomized double-blind clinical trial. J. Periodontal Res. 51 (3), 321–331. 10.1111/jre.12311 26251312

[B56] Tian X. Liu Y. Liu X. Gao S. Sun X. (2019). Glycyrrhizic acid ammonium salt alleviates Concanavalin A-induced immunological liver injury in mice through the regulation of the balance of immune cells and the inhibition of hepatocyte apoptosis. Biomed. Pharmacother. 120, 109481. 10.1016/j.biopha.2019.109481 31586906

[B57] Tuosto L. Cundari E. Gilardini Montani M. S. Piccolella E. (1994). Analysis of susceptibility of mature human T lymphocytes to dexamethasone-induced apoptosis. Eur. J. Immunol. 24 (5), 1061–1065. 10.1002/eji.1830240508 8181517

[B58] Utsunomiya T. Kobayashi M. Herndon D. N. Pollard R. B. Suzuki F. (1999). Effects of glycyrrhizin, an active component of licorice roots, on Candida albicans infection in thermally injured mice. Clin. Exp. Immunol. 116 (2), 291–298. 10.1046/j.1365-2249.1999.00890.x 10337021 PMC1905282

[B59] Wakabayashi A. Shimizu M. Shinya E. Takahashi H. (2018). HMGB1 released from intestinal epithelia damaged by cholera toxin adjuvant contributes to activation of mucosal dendritic cells and induction of intestinal cytotoxic T lymphocytes and IgA. Cell Death Dis. 9 (6), 631. 10.1038/s41419-018-0665-z 29795370 PMC5967345

[B60] Wang W. Chapman N. M. Zhang B. Li M. Fan M. Laribee R. N. (2019). Upregulation of PD-L1 via HMGB1-activated IRF3 and NF-κB contributes to UV radiation-induced immune suppression. Cancer Res. 79 (11), 2909–2922. 10.1158/0008-5472.CAN-18-3134 30737234 PMC6548650

[B61] Wang Y. Zhang H. Chen Q. Jiao F. Shi C. Pei M. (2020). TNF-α/HMGB1 inflammation signalling pathway regulates pyroptosis during liver failure and acute kidney injury. Cell Prolif. 53 (6), e12829. 10.1111/cpr.12829 32419317 PMC7309595

[B62] Wu H. Wu T. Hua W. Dong X. Gao Y. Zhao X. (2015). PGE2 receptor agonist misoprostol protects brain against intracerebral hemorrhage in mice. Neurobiol. Aging 36 (3), 1439–1450. 10.1016/j.neurobiolaging.2014.12.029 25623334 PMC4417504

[B63] Wu X. Wang W. Chen Y. Liu X. Wang J. Qin X. (2018). Glycyrrhizin suppresses the growth of human NSCLC cell line HCC827 by downregulating HMGB1 level. Biomed. Res. Int. 2018, 1–7. 10.1155/2018/6916797 PMC5820661 29568761

[B64] Xiao X. Huang S. Chen S. Wang Y. Sun Q. Xu X. (2021). Mechanisms of cytokine release syndrome and neurotoxicity of CAR T-cell therapy and associated prevention and management strategies. J. Exp. Clin. Cancer Res. 40 (1), 367. 10.1186/s13046-021-02148-6 34794490 PMC8600921

[B65] Xu H. Z. Li T. F. Wang C. Ma Y. Liu Y. Zheng M. Y. (2021). Synergy of nanodiamond-doxorubicin conjugates and PD-L1 blockade effectively turns tumor-associated macrophages against tumor cells. J. Nanobiotechnology 19 (1), 268. 10.1186/s12951-021-01017-w 34488792 PMC8422639

[B66] Xu M. Gong L. Wang B. Wu Y. Wang Y. Mei X. (2018b). Glycyrrhizin attenuates *Salmonella enterica* serovar typhimurium infection: New insights into its protective mechanism. Front. Immunol. 9, 2321. 10.3389/fimmu.2018.02321 30459751 PMC6232675

[B67] Xu M. Min Z. Wei Y. (2018a). Glycyrrhizin inhibits IFN-gamma-induced CXCL10 by suppressing the JAK/STAT1 signal pathway in HaCaT cells. Xi Bao Yu Fen Zi Mian Yi Xue Za Zhi 34 (8), 708–713. 30384869

[B68] Xu N. Yang X. F. Xue S. L. Tan J. W. Li M. H. Ye J. (2022). Ruxolitinib reduces severe CRS response by suspending CAR T cell function instead of damaging CAR T cells. Biochem. Biophys. Res. Commun. 595, 54–61. 10.1016/j.bbrc.2022.01.070 35101664

[B69] Yanagawa Y. Ogura M. Fujimoto E. Shono S. Okuda E. (2004). Effects and cost of glycyrrhizin in the treatment of upper respiratory tract infections in members of the Japanese maritime self-defense force: Preliminary report of a prospective, randomized, double-blind, controlled, parallel-group, alternate-day treatment assignment clinical trial. Curr. Ther. Res. Clin. Exp. 65 (1), 26–33. 10.1016/S0011-393X(04)90002-1 24936101 PMC4052969

[B70] Yao L. Sun T. (2019). Glycyrrhizin administration ameliorates Streptococcus aureus-induced acute lung injury. Int. Immunopharmacol. 70, 504–511. 10.1016/j.intimp.2019.02.046 30884430

[B71] Yu W. Pan Z. Zhu Y. An F. Lu Y. (2017). Fumigaclavine C exhibits anti-inflammatory effects by suppressing high mobility group box protein 1 relocation and release. Eur. J. Pharmacol. 812, 234–242. 10.1016/j.ejphar.2017.06.008 28610842

